# Progress in bioleaching: fundamentals and mechanisms of microbial metal sulfide oxidation – part A

**DOI:** 10.1007/s00253-022-12168-7

**Published:** 2022-10-04

**Authors:** Mario Vera, Axel Schippers, Sabrina Hedrich, Wolfgang Sand

**Affiliations:** 1 Instituto de Ingeniería Biológica y Médica, Escuelas de Ingeniería, Medicina y Ciencias Biológicas, Pontificia Universidad Católica de Chile, Santiago, Chile; 2 Departamento de Ingeniería Hidráulica y Ambiental, Escuela de Ingeniería, Pontificia Universidad Católica de Chile, Santiago, Chile; 3 Bundesanstalt für Geowissenschaften und Rohstoffe (BGR), Hannover, Germany; 4 Institute of Biosciences, TU Bergakademie Freiberg, Freiberg, Germany; 5 Faculty of Chemistry, University Duisburg-Essen, Essen, Germany

**Keywords:** Bioleaching, *Acidithiobacillus*, Metal sulfides, Extracellular polymeric substances, Biofilms

## Abstract

**Abstract:**

Bioleaching of metal sulfides is performed by diverse microorganisms. The dissolution of metal sulfides occurs via two chemical pathways, either the thiosulfate or the polysulfide pathway. These are determined by the metal sulfides’ mineralogy and their acid solubility. The microbial cell enables metal sulfide dissolution via oxidation of iron(II) ions and inorganic sulfur compounds. Thereby, the metal sulfide attacking agents iron(III) ions and protons are generated. Cells are active either in a planktonic state or attached to the mineral surface, forming biofilms. This review, as an update of the previous one (Vera et al., 2013a), summarizes some recent discoveries relevant to bioleaching microorganisms, contributing to a better understanding of their lifestyle. These comprise phylogeny, chemical pathways, surface science, biochemistry of iron and sulfur metabolism, anaerobic metabolism, cell–cell communication, molecular biology, and biofilm lifestyle. Recent advances from genetic engineering applied to bioleaching microorganisms will allow in the future to better understand important aspects of their physiology, as well as to open new possibilities for synthetic biology applications of leaching microbial consortia.

**Key points:**

•* Leaching of metal sulfides is strongly enhanced by microorganisms*

•* Biofilm formation and extracellular polymer production influences bioleaching*

•* Cell interactions in mixed bioleaching cultures are key for process optimization*

## Introduction

The application of bioleaching of metal sulfides (MS) and its understanding have evolved over the last decades. The mobilization of metal cations from often almost insoluble minerals in ores by biological acidification, oxidation, and complexation processes is referred to as bioleaching, and its application is termed biomining, being now a worldwide established geobiotechnological process. Biomining is mainly employed for copper, cobalt, nickel, zinc, gold, and uranium. These are extracted either from insoluble sulfides or—in the case of uranium—from oxides. For gold and silver recovery from refractory ores, the activity of leaching microorganisms is applied only to dissolve metal sulfides like arsenopyrite bearing the precious metals prior to cyanidation treatment. For this process, the term bio-oxidation is used because the solubilized metals such as iron and arsenic are not of economic value. The term biomining covers both applied bioleaching and bio-oxidation (Schippers et al. 2014; Johnson 2015; Kaksonen et al. 2018; Johnson et al. 2022). Recent developments in the fields of molecular biology, “omics” techniques, chemical analysis, biofilm research, and nanotechnology have contributed to an improved understanding of this bioprocess. Nevertheless, which processes are actually occurring at the molecular scale at microbe-mineral interfaces is still not fully known.

Unwanted leaching of metal sulfide-containing ores by leaching microorganisms generates acid mine/rock drainage (ARD/AMD). Improved AMD countermeasures can be developed only if the microbe-mineral interactions are understood thoroughly. The (bio)chemical fundamentals of the leaching reactions have been the subject of intensive research in the last decades. In this context, the sulfur chemistry behind the leaching mechanisms has been widely understood (Schippers 2004; Vera et al. 2013a). The “indirect mechanism,” i.e., the non-enzymatic metal sulfide oxidation by iron(III) ions combined with an enzymatic (re)oxidation of the resulting iron(II) ions, is well accepted to explain bioleaching. In addition, two bioleaching modes exist: “contact” and “non-contact” leaching (Sand et al. 2001; Rawlings 2002). Non-contact leaching is basically exerted by planktonic microorganisms, which oxidize iron(II) ions in solution. The resulting iron(III) ions get into contact with a mineral surface, where they are reduced, and the sulfide moiety is oxidized. Thus, iron(II) ions can enter the cycle again. In a strict sense, this represents the previously designated indirect mechanism (Sand et al. 1995). Contact leaching takes into account that cells attach to the surface of sulfide minerals. This means that the electrochemical processes resulting in the dissolution of sulfide minerals take place at the interface between the microbial cell and the mineral sulfide surface. This space is filled with extracellular polymeric substances (EPS), a mixture of polysaccharides, proteins, lipids, and nucleic acids. However, even after several years of research, many open questions remain. In both, contact and non-contact leaching, the microorganisms contribute to mineral dissolution by the generation of the oxidizing agent, the iron(III) ions, and by subsequent oxidation of the released sulfur compounds arising from the metal sulfide to sulfuric acid. To avoid traces of other metals, which may cause defects/instabilities in the crystal lattice, synthetic minerals produced under rigorously defined conditions can be used for systematic studies on the mechanisms (Tributsch and Bennett 1981). Cell-to-cell communication systems of quorum sensing (QS) are present in some leaching bacteria and control biofilm development (Farah et al. 2005; Ruiz et al. 2008; Gonzalez et al. 2013), but their importance for bioleaching processes remains to be understood. Detailed knowledge of the interactions among the microorganisms in leaching environments, including elucidation of interaction mechanisms and identification of still unknown cell–cell communication signals, may be a future option for further process optimization. As a consequence, this review, which is based on the previous ones (Sand et al. 1995, 2001; Rohwerder et al. 2003; Vera et al. 2013a), required revision. While this mini-review part A focusses on microbiology, biofilm formation and bioleaching mechanisms, part B provides an overview on biomining enriched with metal production data (Roberto and Schippers 2022).

## Diversity of bioleaching microorganisms

Microorganisms commonly thriving in bioleaching operations are acidophilic bacteria or archaea, which are capable of metabolizing iron and reduced inorganic sulfur compounds (RISC) and thereby dissolve minerals. They are often accompanied by obligately heterotrophic bacteria and some fungi. These prokaryotes typically thrive at pH values below 3 and within a wide range of temperatures (Johnson and Quatrini 2020). The diversity of microorganisms in bioleaching operations is determined by the process parameters, e.g. temperature, pH, oxygen availability, carbon source, and solid load. At moderate temperatures, bioleaching communities are dominated mostly by mesophilic genera such as the iron(II)-oxidizing *Acidithiobacillus (At.)*, *Acidiphilium*, *Acidocella*, *Acidiferrobacter*, and *Leptospirillum* (*L.*) *ferrooxidans*. At moderately high temperatures (> 40 ℃ up to maximal 60 ℃), *Firmicutes* (*Alicyclobacillus*, *Sulfobacillus*), *Actinobacteria* (*Ferrimicrobium*, *Acidimicrobium*, *Ferrithrix*, “*Acidithiomicrobium*”) as well as *At. caldus* and *Nitrospira* like *L. ferriphilum* are frequently found. Archaea relevant in bioleaching operations at moderate-to-slightly increased temperatures comprise members of the *Thermoplasmatales* (*Ferroplasma*, *Acidiplasma*, and *Cuniculiplasma*). At high temperatures (> 60 ℃), archaea belonging to the *Sulfolobales*, a group of extremely thermophilic, sulfur and iron oxidizers comprising the genera *Sulfolobus*, *Sulfuracidifex*, *Acidianus*, *Metallosphaera*, and *Sulfurisphaera* become dominant. The diversity and properties of several metal sulfide leaching microorganisms and their applications have also been documented by Quatrini and Johnson (2016).

While *At. thiooxidans* (formerly: *Thiobacillus thiooxidans*) and *At. ferrooxidans* (formerly: *Ferrobacillus ferrooxidans*, *Thiobacillus ferrooxidans*) were the first acidophilic, iron/sulfur oxidizers reported, and several new taxa have been defined and described in the last decades with the advances in molecular methods such as Multi Locus Sequence Analysis (MLSA), Next Generation Sequencing (NGS), Average Nucleotide Identity (ANI), in silico DNA hybridization, among others. The order *Acidithiobacillales*, previously belonging to the class *Gammaproteobacteria*, was assigned to the new class *Acidithiobacillia* (Williams and Kelly 2013). Currently, there are five iron(II)-oxidizing *Acidithiobacillus* species (*At. ferrooxidans*, *At. ferridurans*, *At. ferrivorans*, *At. ferriphilus*, and *At. ferrianus*) assigned using additional marker genes, MLSA-based phylogenies, and oligotyping analysis, which had been recognized all as *At. ferrooxidans* before (Amouric et al. 2010; Norris et al. 2020). *At. sulfuruphilus* was recently described as another sulfur-oxidizing *Acidithiobacillus* species, besides *At. thiooxidans*, *At. caldus*, and *At. albertensis* (Falagan et al. 2019). The newly discovered *Acidithiobacillus* species also brought about specific traits useful in bioleaching, such as *At. ferrivorans*, which have been characterized as psychrophile and motile and have predominantly been reported to occur in low-temperature environments (Quatrini and Johnson 2016). Furthermore, *At. ferrphilus* (Falagán and Johnson 2016) is highly osmotolerant (growth at > 1 M MgSO_4_), which has also been reported for *Acidiferrobacter thiooxydans*, a moderate osmophile of the gamma-proteobacterial class (Hallberg et al. 2011).

Recently, a comprehensive in silico study of nearly 100 representative complete and draft genomes of members of *Acidithiobacillia* was done, using analyses of overall genome relatedness indexes, in silico DNA-DNA hybridization, plus phylogenomic reconstruction based on ribosomal proteins, sets of conserved “core” proteins, including proteins from families of metabolic features of interest. New candidate genera proposed are *Fervidacidithiobacillus* containing mesothermophiles including *At. caldus*, *Igneacidithiobacillus* containing several thermotolerant isolates from volcanic areas worldwide, and *Ambacidithiobacillus* containing *At. sulfuriphilus*, one of the deepest branching clades within this taxon*.* These, in addition to the previously validated *Thermithiobacillus* and *Acidithiobacillus,* form a monophyletic clade, sister to *Betaproteobacteria* or *Gammaproteobacteria.* Interestingly, five main clades were clearly distinguished; deep branching ones are exclusively sulfur oxidizers, while the newest one contains the iron-oxidizing *Acidithiobacillia*. Iron oxidation pathways were acquired lately, probably from a gamma-proteobacterium related to the genus *Acidiferrobacter* genus (Hallberg et al. 2011; Issotta et al. 2018). Also, 12 new species have been proposed, requiring further chemotaxonomic studies and validation (Moya-Beltran et al. 2021).

Bioleaching archaea are not as diverse as their bacterial counterparts. The archaea can, however, be distinguished by their temperature preference in bioleaching. The iron oxidizers *Ferroplasma (Fp.) acidiphilum*, “*Fp. acidamarnus*”, *Acidiplasma (Ap). cupricumulans* (formerly: *Fp. cupricumulans*), and *Ap. aeolicum* have temperature optima < 55 ℃ (Quatrini and Johnson 2016). *Ap. cupricumulans* and *Fp. acidiphilum* have a remarkable dominance in bioleaching operations at moderate-to-slightly increased temperatures (Rawlings and Johnson 2007). The first heterotrophic acidophile isolated was *Thermoplasma acidophilum*, which is also a moderate thermophile and the first acidophilic archaeum formally described. Later on, the discovery of acidophilic Thermoproteota with the genera *Sulfolobus* and *Acidianus* followed. Recently, also a member of the mesophilic and exclusively organotrophic genus *Cuniculiplasma* has been reported in bioleaching systems (Golyshina et al. 2016). Thermophilic archaea often achieve much better metal extraction efficiencies than mesophiles at temperatures up to 85 ℃. They are, however, sensitive to high pulp densities. The first thermophiles observed for mineral sulfide bioleaching were the *Acidianus* species, with the well-known species *Acidianus brierleyi*. Most bioleaching operations at about 70 ℃ are dominated by *Sulfolobus metallicus*, which has been renamed recently as *Sulfuracidifex metallicus* (Itoh et al. 2020). At temperatures above 75 ℃, other archaea, such as *Metallosphaera sedula* and several so far unnamed archaea, are dominant and have been observed to leach chalcopyrite efficiently at temperatures as high as 90 ℃ (Norris 2007). Recently, the novel species *Metallosphaera javensis* has been described (Hofmann et al. 2022).

Leaching microorganisms, such as *Acidithiobacillus* spp., *Leptospirillum* spp., and *Ferroplasma* spp., are chemolithoautotrophs metabolizing iron or sulfur compounds and assimilating carbon, whereas mixotrophic (e.g., *Sulfobacillus* spp.) or (facultative) heterotrophic microorganisms, like *Acidiphilium* spp., use organic carbon compounds besides iron(II) ions and reduced inorganic sulfur compounds (RISC) (Johnson and Quatrini 2020). Most of the mixotrophic and heterotrophic microorganisms in bioleaching systems are capable of either switching quickly their metabolism or even living on very low amounts of organics, e.g. extracellular polymeric substances excreted from primary producers in the system (Okibe et al. 2003).

Besides the dominant iron and sulfur metabolism in acidophiles, several of these organisms are also capable of using hydrogen as an electron donor either coupled to oxygen reduction or to the reduction of iron(III) ions or organic compounds under anaerobic conditions (Hedrich and Johnson 2013; Kucera et al. 2020). Anaerobic growth of several organisms is conducted by iron(III) ions reduction coupled to the oxidation of sulfur, hydrogen, or organic compounds and has been studied intensively for *At. ferrooxidans* (Johnson et al. 2012). *At. ferroxidans* and *S. thermosulfidooxidans* have been reported as the most efficient and dominant bacteria in reductive bioleaching of oxidized ores, whereas *At. thiooxidans* dominates at very low pH contributing to the reductive dissolution of iron(III) ions minerals (Marrero et al. 2020; Malik and Hedrich 2022). Furthermore, bioleaching microorganisms are adapted to thrive under low phosphate availability, due to precipitation in iron-rich environments, by scavenging traces of inorganic phosphate and using alternative phosphorous sources such as phosphonates (Vera et al. 2008). Their extreme tolerance to several toxic metals (e.g. copper, cadmium, zinc, nickel) and metalloids (e.g. arsenic) is based on several physiological and genetic adaptations such as extracellular metal binding elements, a diversity of defense systems against reactive oxygen species (ROS), and specific metal detoxifying systems, e.g. copper chaperones, as well as detoxification copper pumps (Martinez-Bussenius et al. 2016). In addition, several bioleaching species have been classified as polyphosphate accumulating organisms (PAO) due to their ability to store huge amounts of inorganic polyphosphates (polyP). Storage occurs in bioleaching bacteria and archaea and is also postulated as a contributing factor to their high resistance against copper ions (Alvarez and Jerez 2004; Orell et al. 2012).

Their capability of thriving under aerobic and anaerobic conditions, using various energy sources and being tolerant to high load of metals and low nutrient availability, makes acidophilic microorganisms unique for various bioleaching processes and broad applications also in mine water remediation. Challenges in the mining industry regarding ore grade, presence of toxic trace metals, and water scarcity for processing have induced bioprospecting for microorganisms that are tolerant toward increased loads of toxic elements and salinity, but also to extreme bioprocessing conditions such as low pH and high temperature. Studies have shown that mixed cultures improve bioleaching efficiency due to synergistic effects between chemoautotrophic and heterotrophic microorganisms. Furthermore, bioleaching cultures often show shifts in community composition upon changes in chemical and process parameters such as increasing redox potential, low pH, high metal load, increased availability of organic carbon, or presence of different metal sulfides as a substratum (Watling et al. 2013; Hedrich et al. 2016).

Since industrial bioleaching processes are non-sterile, the appearance of indigenous microorganisms in the microbial communities also in lab-scale bioleaching has been observed, leading to the discovery of novel and specially adapted microorganisms for bioleaching processes (Martinez et al. 2015; Kaksonen et al. 2020; Zhang et al. 2021). The application of halotolerant microorganisms in bioleaching became more relevant in the last decade, e.g., for bioleaching in regions where freshwater is scarce or for processes where chloride might enhance the leaching efficiency (Roberto and Schippers 2022). Most acidophiles are inhibited under saline conditions (i.e. seawater chloride concentration), with the exception of a few halotolerant leaching organisms. Bioprospecting has led to the discovery of halotolerant *Acidihalobacter* spp., capable of sulfide ore leaching at high chloride concentrations (Khaleque et al. 2019). Nevertheless, it is well known that microorganisms can be adapted to harsh conditions by repeated cultivation under increasing selection pressure in the laboratory, which is referred to as adaptive laboratory evolution (ALE) (Kaksonen et al. 2020). The main osmoadaptative response mechanisms against the stress caused by chloride consist of the biosynthesis of compatible solutes, such as ectoine, hydroxyectoine, betaine, or threaholse, as well as increased uptake of potassium ions. It is interesting to mention that chloride inhibition depends on the species/strain and its precultivation conditions. For example, in *S. thermosulfidooxidans*, complete inhibition of iron oxidation was achieved at NaCl concentrations of 525 mM, 725 mM, and 800 mM when cells were previously grown on iron(II) ions, tetrathionate, or pyrite, respectively. The addition of NaCl also resulted in increased oxygen respiration, and under these conditions, the addition of small amounts of yeast extract also enhanced copper dissolution from chalcopyrite (Huynh et al. 2020). The response of *L. ferriphilum* against acidification caused by chloride ions also involves an increase in oxygen consumption, and its transcriptional response showed an increase in thioredoxin and alkyl-peroxidase activities (Rivera-Araya et al. 2019). Mixed cultures containing *Am. ferrooxidans*, *S. sibiricus*, *At. caldus*, and *Acidiplasma* sp. were shown to leach chalcopyrite at salinities of 0.5 or 2% NaCl. Under these conditions, especially the latter one, chloride-driven inhibition of iron oxidizers was observed, and redox potentials were lower in comparison to growth in freshwater conditions, leading to an increase of chalcopyrite leaching due to the synergistic action of chloride ions and sulfur oxidizers (Noguchi and Okibe 2020).

Although genetically modified organisms are not allowed to be applied in biomining, the robustness and tolerance to harsh conditions may still be subjected to improvements in order to enhance the bioleaching process. The overexpression of an endogenous glutathione synthase encoding gene in *At. ferrooxidans*^T^ has been recently reported. Interestingly, the enhancement of the intracellular antioxidant glutathione led to an increased halotolerance as recombinant *At. ferrooxidans*^T^ cells were able to grow at up to 200 mM NaCl and oxidize iron(II) at lower pH in comparison to the wild-type strain (Yuta et al. 2021). Interestingly, iron(II) oxidative capabilities of *At. ferrooxidans* (see further) have been subjected to improvements by genetic engineering. The constitutive overexpression of Rus resulted in enhanced iron oxidation activity of strain ATCC 19859 (Liu et al. 2011). Recently, Rus overexpression resulted in the enhancement of microbially influenced corrosion on stainless steel by recombinant *At. ferrooxidans*^T^ cells (Inaba et al. 2020).

## Microbe/mineral interactions—initial cell attachment

In general, leaching bacteria seem to grow attached to the surfaces of mineral sulfides. In case of not limiting surface space, some microorganisms with good attachment capabilities to mineral surfaces exhibit attachment of up to 80–90% of the planktonic cells within less than one day (Gehrke et al. 1998; Harneit et al. 2006). It is quite obvious that cell attachment depends on the strain, its precultivation conditions, as well as the presence of other, in some cases, primary colonizers (i.e. iron oxidizers) on mineral surfaces (Bellenberg et al. 2014). Interestingly, quite a few cells remain planktonically living, and the reason for this has not been clarified yet. The attachment process is mediated predominantly by the extracellular polymeric substances (EPS) surrounding the microbial cells. Surface contact due to cell attachment stimulates EPS production (Vandevivere and Kirchman 1993; Bellenberg et al. 2012). The EPS of *At. ferriphilus* strain R1 (formerly *At. ferrooxidans* R1) grown on pyrite are containing the sugars glucose, rhamnose, fucose, xylose, mannose, C12–C20 saturated fatty acids, glucuronic acid, and iron(III) ions (Gehrke et al. 1998). Attachment of the cells to mineral ores is a result of electrostatic interactions between the positively charged cell surfaces (Dong et al. 2022) with the negatively charged pyrite surface (at pH 2 in sulfuric acid solution) (Solari et al. 1992; Blake et al. 1994). Hydrophobic interactions, which seem to be involved too, are considered of reduced impact on the attachment of cells of *At. ferriphilus* R1 to mineral sulfides (Gehrke et al. 1998; Sampson et al. 2000). In contrast, if *At. ferrooxidans* or *At. ferriphilus* cells have been pregrown on elemental sulfur, they attach only to a very limited extent to FeS_2_. This seems to be a consequence of a considerably modified EPS composition if comparing the latter with those grown on pyrite. EPS of cells grown on sulfur contain considerably less sugars and uronic acids, but significantly more fatty acids than the EPS of pyrite-grown cells. The total lack of iron(III) or other anions in the EPS is the most important difference between these EPS. Consequently, in the case of sulfur, hydrophobic interactions seem to be more relevant for an attachment of cells of *At. ferrooxidans* (Gehrke et al. 1998). Experimental work with an atomic force microscope, which had been equipped with specially modified cantilevers (holding pieces of natural metal sulfide crystals or immobilized cells instead of the usual silicone nitride tips), indicates a specifically increased attachment force of the EPS from iron-grown *Sulfobacillus* cells to pyrite if compared to other mineral substrates (Diao et al. 2014; Li et al. 2019).

**Fig. 1:**
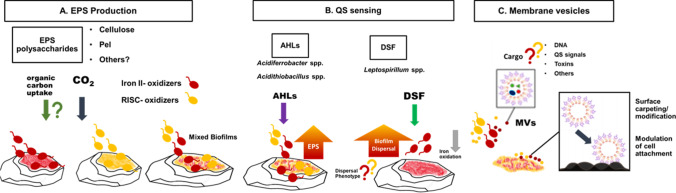
Summary of known aspects and future research challenges on bioleaching biofilms. (**A**) EPS extracellular polysaccharides diversity. Gene machineries for the biosynthesis of cellulose and PEL extracellular polysaccharides have been identified in several strains, and evidence point out that organic carbon, especially monosaccharides, could be uptaken for EPS biosynthesis. (**B**) Quorum sensing (QS): QS type I systems and the presence of N-acyl homoserine lactones (AHL) have been shown to exist in *Acidithiobacillus* and *Acidiferrobacter*, controlling biofilm formation and EPS biosynthesis. Diffusible signal factors (DSF) type molecules have been detected in *Leptospirillum*. Their dynamics in mixed-species cultures and biofilms, as well as the enhancement of dispersion phenotypes, have not yet been clarified. (**C**) “Blebbing”: membrane vesicles (MV) have been detected in several acidophiles. The composition of their cargo, their roles in cell-signaling, surface carpeting/conditioning, and potential inhibitory and “killer” activities remain to be discovered

The molecular mechanisms, which leaching bacteria use to adapt the composition and amount of their EPS to a new growth substrate (planktonic cells grown with soluble substrates such as iron(II) sulfate almost do not produce EPS), remain unclear and need to be elucidated. The attachment sites on mineral surfaces and how the cells can detect/sense these are still open questions and will need attention in the future. Some authors indicate in their publications (Ohmura et al. 1993; Sanhueza et al. 1999; Edwards et al. 2001; Buetti-Dinh et al. 2020) that an attachment of cells to metal sulfide surfaces does not occur randomly (Fig. 1). For example, images obtained by atomic force microscopy (AFM) as well as confocal laser microscopy (CLSM) show that *At. ferrooxidans* cells obviously preferentially attach to sites exhibiting visibly surface imperfections (scratches, etc.). It seems likely that an attachment to surface areas with obvious defects is favored. In addition, sessile cells seem to orient themselves along “crystallographic axes,” as can be derived from the morphology of resulting corrosion pits (Rodriguez-Leiva and Tributsch 1988). Whereas an adhesion to scratches on surfaces can be explained simply by an enhancement of the available contact area, “crystallographic axes” are often not related to changes in surface topography. Therefore, attachment at specific sites on a mineral surface should be in principal related to different attractants. These are probably caused by charge imbalances existing on the mineral surface, like defect sites (S-deficient, Fe^3+^ bearing sites, or vice versa). As a consequence, the number of attachment sites is limited, where cells could start the dissolution of a mineral. Various strains of *At. ferrooxidans* and *L. ferrooxidans* obviously possess a chemosensory system – chemotaxis – which allows them to react positively to the presence of gradients of iron(II)/(III) ions, thiosulfate, etc. (Acuña et al. 1992; Meyer et al. 2002). These compounds are known to be key compounds occurring at the sites of metal sulfide dissolution (Fig. 1). From the point of electrochemistry, the dissolution occurs at local anodes. As a result, iron(II) ions and thiosulfate get in the solution (in the case of FeS_2_); an excellent review on the anodic and cathodic reactions is given by Rimstidt and Vaughan (2003). Most likely, these local anodes are the sites to which the cells are attracted by chemotaxis. Such anodes and cathodes are caused by mineral imperfections of the FeS_2_ crystal lattice, probably, where the iron to sulfur stoichiometry is not exactly 1 to 2, as it is defined for ideal pyrite crystals. Such variations can be a result of variations of the temperature during crystal genesis (causing forms of amorphous up to highly crystalline structures as has been shown for synthetic FeS_2_ (Sanhueza et al. 1999)). Furthermore, inclusions of other metal atoms such as Tl, Sb, or As during the crystallization process result in different chemical properties of the resulting mineral (Soltani Dehnavi et al. 2018). These variabilities of FeS_2_ phases are probably also responsible for the different FeS_2_ oxidation rates published in the literature for bioleaching experiments with FeS_2_ of different origins (Rimstidt and Vaughan 2003).

**Fig. 2:**
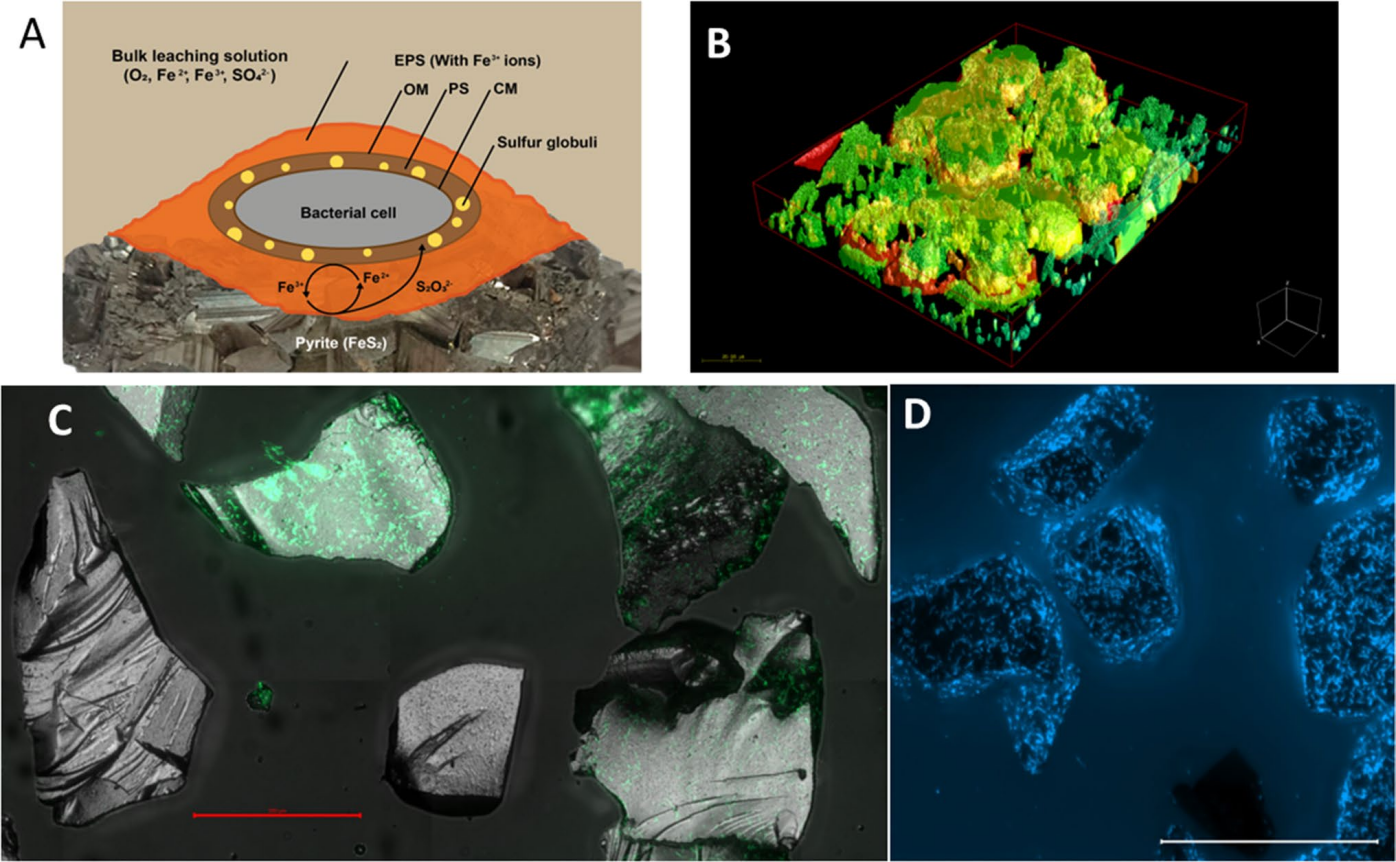
(**A**) Model for contact leaching catalyzed by a cell of *At. ferrooxidans* (from Sand et al. 1995), modified. Overview showing the bacterial cell embedded in its EPS attached to pyrite. CM, cytoplasmic membrane; PS, periplasmic space; OM, outer membrane. (**B**) Mature biofilm formed by *Acidithiobacillus caldus* on sulfur prills after 12 days of incubation. Cells were double stained with FITC-labeled WGA lectin (green) for glycoconjugates and Syto 59 (red, for nucleic acids). Surface protection with merged channels is shown. The size bar indicates 20 µm. (**C**) Epifluorescence microscopy (EFM) coupled with light reflection composed an image of a mixed culture of *At. caldus* and *L. ferrooxidans*, after 48 h biofilm formation on pyrite. Cells were stained with Syto 9 (DNA binding). (**D**) Axenic culture of *Acidiferrobacter* sp. strain SP3 forming biofilms on pyrite after 72 h of incubation. Cells were stained with DAPI (DNA binding). Size bars in (C) and (D) represent 100 µm

Experiments for cell attachment using an AFM equipped for Kelvin Probe Force Mapping (Vera et al. 2013a) indicate for cells of *L. ferrooxidans*, which are attached to a pyrite surface, that they are more negatively charged (about 100–200 mV) than the surrounding surface. Little et al. (2000) have tested the sites for attachment of sulfate-reducing bacteria using steel surfaces. They described that the cells attached in the immediate vicinity (nanometer range) of the anode. The consequence of such bacterial attachment is that anode and cathode are becoming permanent (manifest), which allows steel dissolution to commence. This observation can be applied as well to the bioleaching of metal sulfides, especially since anodic and cathodic reactions are described well for pyrite (Rimstidt and Vaughan 2003). To summarize, in our understanding of microbe-mineral interactions, the cells are attracted to electrically charged (perhaps transient) sites of mineral dissolution due to chemotactic attraction to liberated iron ions and RISCs. As a result, the cells attach and cause the anodes and cathodes on the metal sulfide surface to become permanent. The dissolution reactions take place in the EPS film (Fig. 2). This layer fills the tiny distance between the cell’s outer membrane and the surface layer of the metal sulfides. Thus, it can be considered as a special reaction space. Rodriguez-Leiva and Tributsch (1988) provided evidence in their work that the distance between these surfaces may range between 10 and 100 nm. In the case of metal sulfides such as pyrite, which is a metal sulfide needing an oxidizing attack on its crystal structure by aqueous iron(III) ion complexes (Moses et al. 1987), the EPS-harboring iron(III) ions might play a role as well. However, this has recently been challenged, and iron(III) ions are unlikely to be complexed in the EPS of strain *At. ferrooxidans* Acfo 1 grown on iron(II) ions (Dong et al. 2022), which would be in contrast to our previous assumptions (Sand et al. 2001; Vera et al. 2013a). The *At. ferrooxidans* EPS thickness was estimated for iron(II) grown cells by in vivo AFM to be 28.7 nm (± 13.5). The polymer density calculations estimated that this bacterium has 51,000 to 105,000 exopolymer molecules on its outer surface, a value 20–30 times lower than that for *E. coli* (Taylor and Lower 2008). The EPS thickness values for sulfur or pyrite-grown cells remain to be elucidated. Presumably, these will be much higher than the above-mentioned ones since it is already known that EPS levels increase by more than 20 times if the bacteria are grown with these substrates (Gehrke et al. 1998). However, we are still assuming that the iron(III) ions on the cell surface (e.g. in the EPS) are reduced after getting in contact with the mineral surface. Consequently, the iron(III) ions in the EPS have to be exposed to the pyrite surface within less than a distance of 2 nm (in order to be reducible by tunneling electrons). If iron(II) ions diffuse toward the outer membrane, they will be (re)oxidized by the enzymatic system of the cells to iron(III) ions. This hypothesis of an electron transfer via iron cycling is providing a likely explanation of the microbe-mineral interactions on metal sulfide surfaces based on electrochemical processes, as has been described for pyrite oxidation (Rimstidt and Vaughan 2003) and is in agreement with the bioleaching mechanisms (Schippers and Sand 1999) described below.

## Biofilm formation and molecular controls

Experiments with *At. ferrooxidans*^T^ growing on floating filters show that the addition of glucose or galactose enhanced capsular polysaccharide biosynthesis. EPS formation was also induced by cultivation under P*i* starvation. This enhancement was observed by confocal laser scanning microscopy (CLSM) using fluorescently labeled lectin-binding assays (FLBA) with Concanavalin A (Con A) lectin, which binds to α-mannose and α-glucose linkages (Bellenberg et al. 2012). Interestingly, the degree of cell attachment to pyritic ores by iron-oxidizing species of *Acidithiobacillus* can also be manipulated under laboratory conditions. The influence of precultivation conditions, pH, ionic strength of the medium, and nutritional supplementation seem to play a role. The addition of sodium glucuronate (at a subinhibitory concentration of 1 mM) increased cell attachment and pyrite dissolution in *At. ferrooxidans*^T^ and *At. ferridurans* SS3 (Bellenberg et al. 2015). This effect has been explained by an enhanced initial attachment due to the increased glucuronic acid residues present in EPS. Although sugars alone do not support the growth of acidithiobacilli, this and other reports strongly suggest that sugars may be taken up and used as polysaccharide building blocks for biosynthesis of extracellular polymers in *Acidithiobacillus* strains. Otherwise, the sugars may just increase the cell metabolism in generell and cell growth, as reported (Tabita and Lundgren 1971). This highlights the importance of mixed communities, in which interdependent nutritional relationships between chemolithotrophic and heterotrophic microorganisms, e.g. *Acidiphilium* sp., exist (Hallmann et al. 1993; Johnson 2001). Another role of EPS, in addition to the mediation of cell attachment to pyrite, may be to act as a scavenger of reactive oxygen species (ROS), which are largely produced at the interface of the mineral due to the presence of lattice-bound Fe^3+^ (Bellenberg et al. 2019).

The biosynthetic capacities for extracellular polysaccharides have been explored to a certain extent in acidophiles. OMIC studies showed that gene machineries for capsular polysaccharides of the Wzy/Kps are present in acidithiobacilli (Vera et al. 2013b; Bellenberg et al. 2015). In addition, genes encoding for cellulose synthase (*bcs* operon) and UDP-D-galacturonate biosynthesis are present in several acidithiobacilli strains, although a cellulose synthase has not been found to be encoded in *At. ferrooxidans*^T^. *At. ferrivorans* CF27 also possess the *fcl* gene, probably involved in GDP-L-fucose biosynthesis (Talla et al. 2014). The sulfur oxidizers *At. thiooxidans* and *At. caldus* possess genes encoding for PEL-like extracellular polysaccharide (Díaz et al. 2018). The construction of an *At. thioooxidans* Δ*pelD* null-mutant strain revealed that PEL exopolysaccharide is involved in its biofilm architecture since their biofilms were more sensitive to mechanical stress and easily removable from sulfur upon mechanical stress in comparison to wild-type strains (Díaz et al. 2020). The analysis of *L. ferriphilium* DSM 14647^ T^ complete genome sequence showed that cellulose, PEL, and poly-1,6-*N*-acetyl-D-glucosamine (PGA) can be synthesized by this strain. It has been suggested that cellulose can not just to be used as a structural EPS element but also as an intracellular carbon storage component in this strain (Christel et al. 2017). Despite all the information presented, there is still a severe lack of information on the complete repertoire of EPS polysaccharides synthesized by bioleaching microorganisms, as well as their modifications upon biofilm formation/switching from/to planktonic states, changes of electron donors, and interactions within mixed communities (Fig. 2). Presumably, these polymers will also possess interesting properties such as water retention, heavy metal binding, and acid resistance and may also contribute to the formation of cell aggregates, which are frequently observed in mixed cultures, especially upon exposure to chalcopyrite concentrates. Several acidophiles are capable to form “blebs.” The formation and release of membrane vesicles is considered conserved phenomena occurring across all domains of life. Their sizes generally range between 50 and 200 nm. Membrane vesicles allow several molecules to be trafficked under a protected environment between neighboring cells. These have been found to be produced in acidophilic archaea, as well as in *Acidiphilium cryptum* (Küsel et al. 1999) and in *At. thiooxidans* (Knickerbocker et al. 2000), suggesting their role in cell colonization on sulfur due to their amphiphilic properties. Their mechanisms of generation and diversity of cargo remain to be elucidated (Fig. 2).

In comparison to pure cultures, microbial biofilm communities are characterized by their robustness and high viability due to the formation of several types of interactions between their constituent species. Mutualistic, synergistic, as well as antagonistic and strong competition, are the main interactions suggested to occur in bioleaching environments (Quatrini and Johnson 2016, 2018). The sum of biofilm interactions leads to “emergent properties,” which are not shown by the individual community members in axenic cultivation systems (Flemming et al. 2016). Bioleaching microbial communities generally possess a relatively low diversity of species, a property that makes them ideal models to study biofilm-related emergent properties. In recent years, one of the main features of developments in biohydrometallurgy has been the isolation and study of microbial communities from natural and technogenic ecosystems. In this regard, molecular technologies such as Omics have become relevant (Martinez et al. 2015).

Biofilm formation is controlled by several intricate molecular networks. Among these, quorum sensing (QS) and cyclic diguanylate c-di-GMP are the most known and studied ones in bacteria. Some *Acidithiobacillus* spp. possesses functional auto inducer (AI)-1-type QS systems, involving biosynthesis of middle–long-chain N-acyl-homoserine lactones (AHLs) as AIs; these are synthesized and sensed by Lux-I/R family proteins (Farah et al. 2005; Rivas et al. 2005). The external addition of synthetic long-chain AHLs stimulates biofilm formation and EPS polysaccharides production in *At. ferrooxidans*^T^ (Gonzalez et al. 2013), while the addition of middle chain AHLs resulted in an enhanced biofilm formation of *At. thiooxidans* on sulfur surfaces (Díaz et al. 2020). At least 75 genes have been predicted to be modulated by QS in *At. ferrooxidans*^T^, several of them are likely to be involved in biofilm-related phenotypes (Banderas and Guiliani 2013). AHL production is also exerted by other species. These have been found in *At. ferrivorans* SS3, *At. thiooxidans* DSM 14887^ T^, and two strains of *Acidiferrobacter* spp.; *Leptospirillum* species do not synthesize AHLs but possess LuxR-like receptors and respond to their addition (Bellenberg et al. 2014). Massive bacterial genome analyses have shown that genes encoding LuxR-like receptors exceed by far the amount of synthase genes. These “orphan receptors” could represent fine-tuning systems, allowing cells to sense the presence of several AHL synthesized by microbial neighbors/competing species (Prescott and Decho 2020).

The intracellular second messenger c-di-GMP is also involved in fine-tuning of biofilm formation. This molecule links the sensing of environmental or intracellular signals with signal transduction networks and effectors by binding to a diverse repertoire of effector molecules, which interact with target components, resulting in several phenotypes. It is synthesized by diguanylate cyclases (DGCs) and degraded by phosphodiesterases (PDEs). C-di-GMP pathways have been shown to be functional in *At. ferrooxidans*, where levels of this second messenger were found to be increased in cells grown on solid substrates such as S^0^ or pyrite (Ruiz et al. 2011), and in *At. caldus* ATCC 51756^ T^, where it is involved in the regulation of swarming motility and cell attachment to S^0^ surfaces (Castro et al. 2015). It has also been shown that QS and c-di-GMP are connected since a PelD mutant strain of *At. thiooxidans* displayed changes in its biofilm architecture upon the addition of AHLs, also showing different fluorescent lectin-binding patterns (Díaz et al. 2020). It has recently been shown that in addition to c-di-GMP, several other messenger nucleotides (cAMP, cGMP, c-di-AMP, and (p)ppGpp) are present in the genomes of acidihiobacilli. Their complexity is strongly related to the strain level, as several determinants were found to be encoded in plasmids and mobile genetics elements (MGE) (Moya-Beltrán et al. 2019). The presence of c-di-GMP is not restricted just to *Acidithiobacillus*, as genome analysis of *L. ferriphilum*^T^ and other acidophilic genera such as *Acidiphilium*, *Sulfobacillus*, and *Alicyclobacillus* showed the presence of gene modules for c-di-GMP metabolism (Christel et al. 2017; Castro et al. 2020).

A particular type of QS system is based on “diffusible signal factors” (DSF), a group of signals that comprises cis-2-unsaturated fatty acids of different chain lengths and branching. In many Gram-negative bacterial species, their signaling involves changes in c-di-GMP levels, leading to various biological responses such as interspecies/interkingdom biofilm dispersal, motility, virulence, and antibiotic resistance (Zhou et al. 2017). The main described compounds are DSF ((Z)-11-methyl-2-dodecenoic acid) and BDSF ((Z)-2-dodecenoic acid). *L. ferriphilum*^T^ and *L. ferrooxidans* possess complete DSF signaling systems, including DSF synthase (*rpfF*), the corresponding two-component signal recognition system, composed of a signal transduction sensor kinase (*rpfC*), and the response regulator (*rpfG*) (Christel et al. 2017; Bellenberg et al. 2018). These genes are expressed in *L. ferriphilum*, and increased *rpf* gene RNA transcripts are present in continuous iron(II) grown cultures and in batch chalcopyrite cultures (Buetti-Dinh et al. 2020). Interestingly, DSF synthase showed high expression levels in the axenic planktonic cell subpopulations but also in mixed cultures with *S. thermosulfidooxidans*^T^ (Bellenberg et al. 2018). The external addition of DSF and BDSF signal compounds exerts a strong inhibitory effect on the metabolic activity of bioleaching bacteria and their biofilm-forming capability by inhibiting iron(II) oxidation of *At. ferrooxidans* ATCC 53993, *At. ferridurans* ATCC 33020^T^, *L. ferrooxidans* DSM 2705^T^, and *Acidiferrobacter* sp. SPIII/3 DSM 27195. Recently, DSF family compounds have been identified in batch pyrite cultures of *L. ferriphilum*^T^ and *L. ferrooxidans*^T^, especially at later stages of cultivation (Bellenberg et al. 2021). All these recent findings suggest that interspecies communication based on QS/ diffusible signaling molecules or even toxins may be diverse in bioleaching bacteria. Its relevance for optimization of bioleaching processes, where mixed cultures are used, remains to be elucidated.

Progress has also been made in biofilm visualization by the use of labeled lectins coupled to epifluorescence microscopy (EFM) or confocal laser scanning microscopy (CLSM). In general, bioleaching microorganisms form monolayer biofilms on metal sulfide surfaces (i.e., pyrite/chalcopyrite), while more structured ones appear on sulfur surfaces (Fig. 1). Fluorescent lectin-binding analysis (FLBA) showed several glycoconjugates to be present upon biofilm formation in thermophilic archaea and bacteria. Positive signals of lectin-binding fucose, glucose, galactose, mannose, N-acetyl glucosamine (GlcNAc), and N-acetyl galactosamine (GalNAc) were detected in these biofilms. FLBA patterns of biofilm cells on pyrite or sulfur were different, suggesting strain- and substrate-related changes in the EPS glycoconjugates/glycoproteins (Zhang et al. 2014; Neu and Kuhlicke 2017). Time series of biofilm pictures revealed that *S. metallicus*^T^ biofilm cells are embedded in a flexible EPS matrix (Zhang et al. 2015).

As biofilm formation of bioleaching microorganisms does not occur randomly (Fig. 1), a natural bias occurs when just a few images are analyzed. Therefore, massive image acquisition and analysis pipelines are necessary for quantitative biofilm studies. Cell attachment on pyrite and chalcopyrite surfaces with axenic and mixed cultures of *At. caldus*^T^, *L. ferriphilum*^T^, and *S. thermosulfidooxidans*^T^ was imaged and quantified, confirming that *L. ferriphilum*^T^ shows the highest biofilm growth. Biofilm reduction was also quantified by this methodology upon the addition of synthetic DSF family signal compounds (Bellenberg et al. 2018). In addition, deep neural networks have been applied to classify biofilm colonization patterns of three different bioleaching bacterial species on chalcopyrite particles. Around 500 images per category were shown to be sufficient for a highly efficient computational analysis of the biofilm bacterial composition, reaching 90% accuracy (Buetti-Dinh et al. 2019).

## Bioleaching pathways

Bioleaching is the dissolution of ore or another solid material by chemical reactions catalyzed by microorganisms. It can be differentiated based on the chemical processes described in detail (Glombitza and Reichel 2014).

1. Oxidative bioleaching
  Oxidative bioleaching is industrially applied for metal recovery from metal sulfides described as biomining (see review part B, Roberto and Schippers 2022). Iron(II) ions and sulfur compound-oxidizing bacteria and archaea dissolve metal sulfides by using molecular oxygen as an electron acceptor in a sequence of chemical reactions described as thiosulfate and polysulfide pathways (see below).
2. Acid bioleaching
  Acid leaching is applied in hydrometallurgy by using inorganic acids for the dissolution of ores, e.g., oxide ores such as limonitic laterites. Sulfuric acid can also be generated by sulfur-oxidizing bacteria or archaea if elemental sulfur is added to the process, or in the case of metal sulfides from the sulfur moiety of the mineral being oxidized. For metal sulfides, acid bioleaching can hardly be differentiated from oxidative bioleaching since their dissolution processes involve both iron(III) ions generated by oxidation reactions and protons (acid).
  Heterotrophic bacteria and fungi produce organic acids, which allow for metal extraction from solids based on acidity and also on the complexation of metals. This kind of bioleaching has been termed “heterotrophic bioleaching.” However, this term is misleading since these microorganisms are heterotrophs, but bioleaching is not a process able to be purely driven by heterotrophic microorganisms in the absence of chemolithotrophs or suitable carbon sources. It may be better described as acid bioleaching by heterotrophs.
3. Reductive bioleaching
  Reductive bioleaching refers to the dissolution of ore or another solid material by a chemical reduction reaction catalyzed by microorganisms. The main reaction is the dissimilatory reduction of iron(III) ions (Malik and Hedrich 2022). The most prominent example is reductive bioleaching of oxide ores such as limonitic laterites (Marrero et al. 2020). However, since elemental sulfur is added as a reducing agent and as well as a source for sulfuric acid, a differentiation from acid bioleaching is difficult in the case of laterite bioleaching. This is described in more detail in part B of this review (Roberto and Schippers 2022). Since several microorganisms including acidophiles are able to couple hydrogen oxidation with iron(III) ions reduction, hydrogen might be an interesting reducing agent for future studies (Malik and Hedrich 2022).

For oxidative bioleaching, a holistic perspective on the role of bacteria in metal sulfide dissolution processes is given in this chapter. Metal sulfides comprise, on the one hand, iron sulfides such as pyrite or pyrrhotite, which generate sulfuric acid by their oxidative dissolution. This can be either beneficial for metal recovery via leaching or detrimental as acid mine/rock drainage. On the other hand, metal sulfides comprise important minerals for base metal mining such as copper, zinc, or lead. During the oxidative dissolution of metal sulfides, various sulfur compounds with an intermediate oxidation state between sulfide (-2) and sulfate (+ 6) occur. Iron(III) ions and molecular oxygen, as well as manganese oxides and nitrate, have been described as oxidants for metal sulfides as well as for intermediate sulfur compounds (Schippers 2004; Yan et al. 2019). Iron- and sulfur-oxidizing bacteria and archaea enzymatically accelerate most of the chemical oxidation reactions, which are described as indirect bioleaching for acidophiles thriving at low pH < 3.

The mechanisms of bioleaching, i.e., the chemical pathways of biological metal sulfide oxidation, have thoroughly been debated in the past. In the earlier literature, “direct” vs. “indirect” bioleaching is described (Rossi 1990; Bosecker 1997; Ehrlich 2016). “Direct” bioleaching has been described as enzymatic metal sulfide oxidation by acidophilic microorganisms being attached to the metal sulfide surface. “Direct” leaching of attached bacteria would mean a direct electron transfer from the sulfide mineral to cell surface compounds (e.g. cytochromes), and further to molecular oxygen. Evidence for this direct electron transfer was provided by bioleaching experiments with *At. ferrooxidans* in “iron-free” medium and using metal sulfides such as synthetic covellite (CuS) (Ehrlich 2016). These experiments have to be carefully interpreted since iron is an essential nutrient, and at least traces of iron in the cells or even bound in their extracellular polymeric substances (EPS) always occur and cannot be avoided.

Direct electron transfer between minerals and bacterial cells has been demonstrated for the neutrophilic, organotrophic, iron(III) ions-reducer *Geobacter sulfurreducens*, which has electron transporting pili (nanowires) on the cell surface, allowing the transfer of electrons to iron oxides. Thereby, it dissolves them via their chemical reduction (Reguera et al. 2005). However, such nanowires have not been found on cells of acidophiles.

Since neither “iron-free” bioleaching nor electron transporting pili have been confirmed for acidophiles, “direct” bioleaching may not exist. In contrast, “indirect” bioleaching refers to the biological oxidation of Fe(II) to Fe(III), the latter being the chemical oxidant for the metal sulfide. Iron ions may occur in solution, adsorbed to the mineral surface, or bound in EPS of bioleaching cells (Sand et al. 1995, 2001). Fe(II) as well as sulfur compounds as products of the incomplete oxidation of the sulfur moiety of metal sulfides may be oxidized either by planktonic cells or by attached cells. For these processes, the terms “non-contact leaching” and “contact leaching,” respectively, and for both processes together, the term “cooperative leaching” have been suggested (Rawlings 2002). However, these terms rather describe the location of bioleaching cells, but they do not tell us anything about the underlying chemical mechanisms of biological metal sulfide dissolution.

Metal sulfides are conductors, semiconductors, or insulators, and their metal and sulfur atoms are bound in the crystal lattice (Vaughan and Craig 1978; Xu and Schoonen 2000; Rimstidt and Vaughan 2003). According to the molecular orbital and valence band theory, the orbitals of single atoms or molecules form electron bands with different energy levels. The metal sulfides FeS_2_ (pyrite), MoS_2_ (molybdenite), and WS_2_ (tungstenite) consist of pairs of sulfur atoms (Vaughan and Craig 1978), which form nonbonding orbitals. Consequently, the valence bands of these metal sulfides are only derived from orbitals of metal atoms, whereas the valence bands of all other metal sulfides are derived from both metal and sulfur orbitals (Borg and Dienes 1992). Thus, the valence bands of FeS_2_, MoS_2_, and WS_2_ do not contribute to the bonding between the metal and the sulfur moiety of the metal sulfide. This fact explains the resistance of these metal sulfides against a proton attack. The bonds can only be broken via multistep electron transfers with an oxidant like the iron(III) ion. For the other metal sulfides, in addition to an oxidant like iron(III) ions, protons can remove electrons from the valence band, causing cleavage of the bonds between the metal and the sulfur moiety of the metal sulfide. Consequently, these metal sulfides are relatively soluble in acid, whereas FeS_2_, MoS_2_, and WS_2_ are insoluble (Crundwell 1988; Sand et al. 2001; Schippers 2004).

Based on the existence of two different groups of metal sulfides, two different metal sulfide oxidation pathways (mechanisms) have been proposed (Schippers and Sand 1999; Sand et al. 2001; Vera et al. 2013a). These mechanisms are able to explain the occurrence of all inorganic sulfur compounds, which have been documented for bioleaching environments. However, chemically pure metal sulfides are not occurring under natural conditions (except if prepared in the lab). Thus, in practice always, mixed minerals are available, causing the generation of mixed reaction products (Sanhueza et al. 1999; Dong et al. 2022) and galvanic coupling (Tanne and Schippers 2021).

**Fig. 3:**
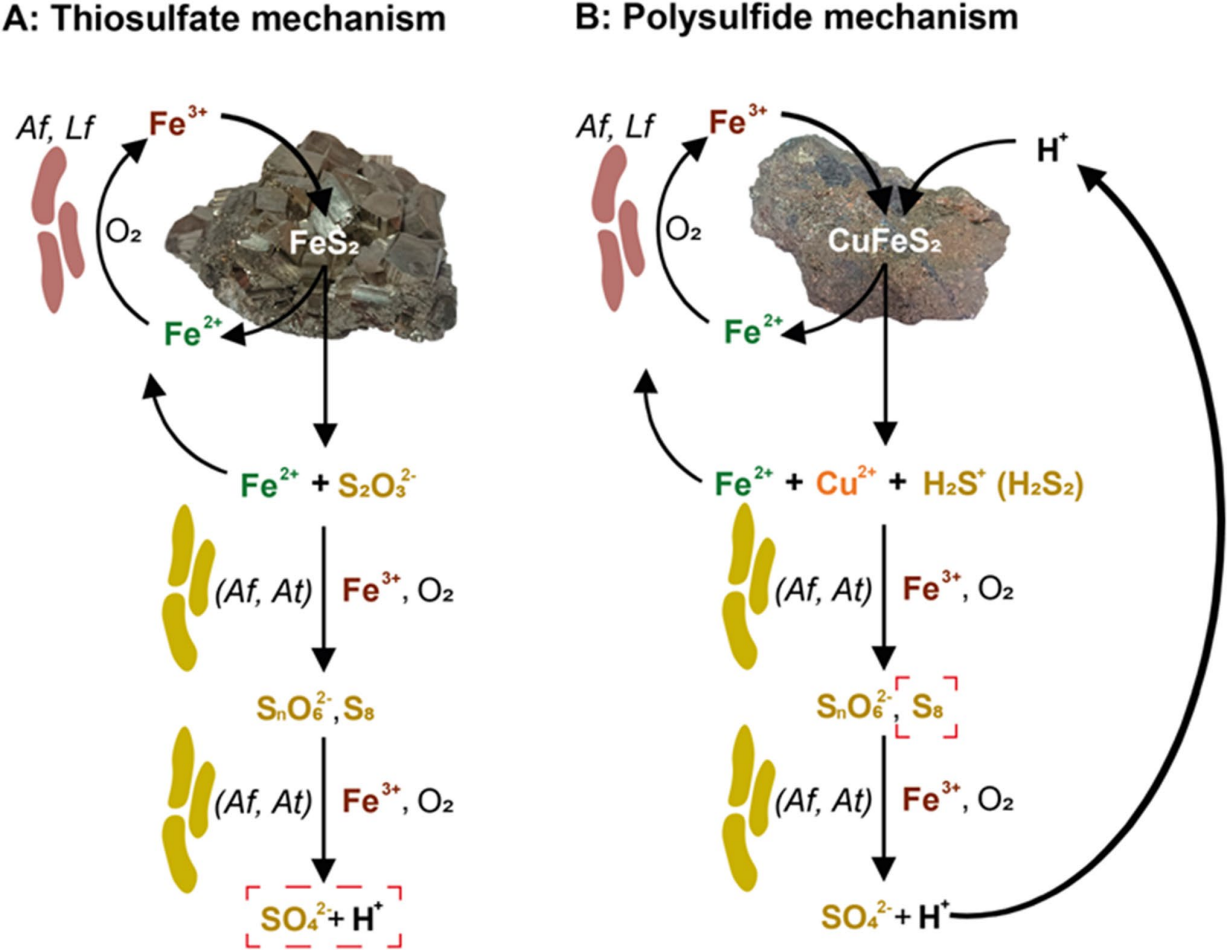
Schematic comparison of the thiosulfate (**A**) and polysulfide (**B**) pathways in (bio)leaching of metal sulfides (from Schippers and Sand (1999), modified). Iron(III) ions attack metal sulfides (MS) by electron extraction and are thereby reduced to iron(II) ions. As a result, the metal sulfide mineral releases metal cations (M.^2+^) and water-soluble intermediary sulfur compounds. Iron(II)-oxidizing bacteria such as *At. ferrooxidans* (Af) and *L. ferrooxidans* (Lf) catalyze the re-oxidation of iron(II) to iron(III) ions in acidic solutions. In the case of acid-soluble metal sulfides (**B**), an additional attack is performed by protons, which can bind valence band electrons of these metal sulfides. The liberated sulfur compounds are oxidized abiotically and by sulfur compound-oxidizing bacteria such as *At. ferrooxidans* and *At. thiooxidans* (At). In the case of mainly abiotic reactions, the contribution of sulfur compound oxidizers is indicated in brackets. The main electron acceptors of oxidation reactions other than the initial iron(III) ion attack on the metal sulfide are given to the right of the arrows. The main reaction products that accumulate in the absence of sulfur compound oxidizers are boxed, i.e., sulfuric acid in (A) and elemental sulfur in (B). The equations given are not stoichiometric. For details, see text and Schippers and Sand (1999) and Schippers (2004)

For pyrite, molybdenite, and tungstenite, the thiosulfate mechanism, and for the other metal sulfides, the polysulfide mechanism has been described (Fig. 3). These pathways are basically indirect, and iron in the EPS layer of mineral attached bioleaching cells might play a dominant role for the metal sulfide dissolution kinetics. The formation of the intermediate sulfur compounds in the two reaction pathways depends on the mineralogy of the metal sulfide and the geochemical conditions in the environment, mainly the pH and the presence of different oxidants (Schippers 2004). Microorganisms play a crucial role in the oxidation of intermediate sulfur compounds, which are formed by the chemical dissolution of the metal sulfides. Under oxic and acidic conditions relevant for bioleaching, microorganisms oxidize Fe(II) to Fe(III) ions, which serve as oxidants for the metal sulfides and as well for most of the intermediate sulfur compounds. Additionally, microorganisms can catalyze enzymatically the oxidation of intermediate sulfur compounds to sulfuric acid.

Support for the relevance of these two different pathways came from measurements of oxygen stable isotopes in the educts and products of pyrite and sphalerite bioleaching experiments with *At. ferrooxidans*. Stoichiometric and isotopic mass balance calculations show that oxygen in the final product sulfate is mainly derived from oxygen in the water and only to a minor extent (~ 10%) from molecular oxygen, consistent with the thiosulfate and polysulfide pathways, respectively. If “direct” bioleaching exists, oxygen in sulfate would be derived from molecular oxygen in contrast to the experimental data, which indicates “indirect” bioleaching via Fe(II)/Fe(III). Some molecular oxygen is only consumed for the biological oxidation of sulfur compounds (Balci et al. 2007, 2012).

## Pyrite and other acid-non-soluble metal sulfides: the thiosulfate pathway

Among metal sulfides, pyrite and its oxidation is most studied (Rimstidt and Vaughan 2003; Schippers 2004) and used as a representative for the three metal sulfides—FeS_2_, MoS_2_, and WS_2_—here. After an initial attack by the oxidant Fe(III) ion, the sulfur moiety of pyrite is oxidized to soluble sulfur intermediates. Moses et al. (1987) and Luther (1987) presented a detailed reaction mechanism for pyrite dissolution by iron(III) ions, in which thiosulfate is the first soluble sulfur intermediate. According to this mechanism, hydrated iron(III) ions oxidize the disulfide moiety of pyrite to a sulfonic acid group by several electron extractions. Due to this transformation, the bonds between iron and the two sulfur atoms are cleaved and hydrated iron(II) ions and thiosulfate are formed. Thiosulfate, as the first soluble sulfur compound intermediate, then, is oxidized almost quantitatively to tetrathionate. Tetrathionate is further degraded to various sulfur compounds, i.e. trithionate, pentathionate, elemental sulfur, and sulfite (Schippers et al. 1996; Schippers 2004; Druschel and Borda 2006). These sulfur compounds are finally oxidized to sulfate in chemical and/or biological reactions. Overall, the thiosulfate pathway can be summarized by the following equations:

$$\begin{array}{ccc}{\mathrm{FeS}}_{2}+6{\mathrm{Fe}}^{3+}+3{\mathrm{H}}_{2}\mathrm{O}& \to & {\mathrm{S}}_{2}{{\mathrm{O}}_{3}}^{2-}+7{\mathrm{Fe}}^{2+}+6{\mathrm{H}}^{+}\end{array}$$

$$\begin{array}{ccc}{\mathrm{S}}_{2}{{\mathrm{O}}_{3}}^{2-}+8{\mathrm{Fe}}^{3+}+5{\mathrm{H}}_{2}\mathrm{O}& \to & {2{\mathrm{SO}}_{4}}^{2-}+8{\mathrm{Fe}}^{2+}+10{\mathrm{H}}^{+}\end{array}$$

The stoichiometry of the thiosulfate pathway has been confirmed in bioleaching experiments with *At. ferrooxidans*, in which the stable isotopes of oxygen and sulfur were determined in the pyrite oxidation reaction products (Balci et al. 2007).

Besides the above-mentioned thiosulfate pathway, other dissolution pathways for pyrite have been suggested (Rimstidt and Vaughan 2003; Druschel and Borda 2006). In the thiosulfate mechanism, thiosulfate is the initial soluble intermediate in the pathway of pyrite oxidation. However, at low pH and especially in the presence of soluble Fe(III) under bioleaching conditions, thiosulfate is hardly detectable, and also the consecutive polythionates can only be detected in low concentrations, which is in contrast to chemical pyrite oxidation at less acidic or even neutral pH, at which thiosulfate and polythionates accumulate (Schippers et al. 1996; Schippers 2004). The release of the two sulfur atoms containing thiosulfate as the initial soluble intermediate is based on the assumption that the strength of the S–S bond in the pyrite crystal lattice is stronger than that of the Fe-S bond. However, the bond strength seems to be dependent on the pH, meaning that the S–S becomes weaker at low pH. As a consequence, the S–S bond might break earlier than the Fe-S bond, which means that sulfite or sulfate would be the initial soluble intermediate, and not thiosulfate (Rimstidt and Vaughan 2003; Druschel and Borda 2006). Vaughan and Coker (2017) argue that depending on the pH, either thiosulfate at higher pH or sulfate at lower pH is released during pyrite oxidation. Recent experimental electrochemical evidence for sulfate rather than thiosulfate release was given for pH 1.8. However, sulfur compounds have not been measured in this study (Borilova et al. 2018). Future studies should address the release of sulfur compounds during pyrite oxidation at different pH values and redox potentials to reveal if other pathways than the thiosulfate mechanism exist for certain bioleaching conditions.

## Most metal sulfides: the polysulfide pathway

In contrast to pyrite oxidation, the metal–sulfur bonds in the acid-soluble metal sulfides can be cleaved before the sulfidic sulfur is oxidized. These metal sulfides like As_2_S_3_ (orpiment), As_4_S_4_ (realgar), CuFeS_2_ (chalcopyrite), FeS (troilite), Fe_7_S_8_ (pyrrhotite), MnS_2_ (hauerite), PbS (galena), and ZnS (sphalerite), thus, can be dissolved by protons. At low pH, the sulfur moiety of these metal sulfides is oxidized mainly to elemental sulfur (Schippers et al. 1996; McGuire et al. 2001). The formation of elemental sulfur via polysulfides is described in a series of reactions for acid-soluble metal sulfides (Schippers and Sand 1999). Polysulfides have been detected during the dissolution of, e.g. Fe_7_S_8_ (Thomas et al. 2001), PbS (Smart et al. 2000), and CuFeS_2_ (Hackl et al. 1995). Because of this sulfur compound intermediate, the oxidation mechanism for acid-soluble metal sulfides has been named the polysulfide mechanism (Schippers and Sand 1999). Elemental sulfur is chemically inert in natural environments, but it can be oxidized biologically to sulfuric acid. Overall, the polysulfide mechanism can be described by the following equations (Schippers and Sand 1999):

$$\begin{array}{cc}\begin{array}{ccc}\mathrm{MS}+{\mathrm{Fe}}^{3+}+{\mathrm{H}}^{+}& \to & {\mathrm{M}}^{2+}+0.5{\mathrm{ H}}_{2}{\mathrm{S}}_{\mathrm{n}}+{\mathrm{Fe}}^{2+}\end{array}& \left(\mathrm{n}\ge 2\right)\end{array}$$

$$\begin{array}{ccc}0.5{\mathrm{H}}_{2}{\mathrm{S}}_{\mathrm{n}}+{\mathrm{Fe}}^{3+}& \to & 0.125{\mathrm{S}}_{8}+{\mathrm{Fe}}^{2+}+{\mathrm{H}}^{+}\end{array}$$

$$\begin{array}{ccc}0.125{\mathrm{S}}_{8}+\mathrm{1.5}{\mathrm{O}}_{2}+{\mathrm{H}}_{2}\mathrm{O}& \to & {{\mathrm{SO}}_{4}}^{2-}+2{\mathrm{H}}^{+}\end{array}$$

The polysulfide pathway is in agreement with the results of bioleaching experiments with *At. ferrooxidans*, in which the stable isotopes of oxygen and sulfur were determined in the products of chalcopyrite and sphalerite oxidation (Thurston et al. 2010; Balci et al. 2012).

## Sulfur chemistry—implications for bioleaching kinetics

In both pathways, the generation of iron(III) ions as an oxidant for metal sulfides via iron(II)-oxidation activity is the main role of acidophilic leaching microorganisms in acidic biotopes (Fig. 3). The redox potential in such acidic environments is determined mainly by the iron(III)/iron(II) ratio in solutions being controlled by the acidophilic iron(II) oxidizers. In addition, acidophilic sulfur oxidizers allow for the oxidation of the intermediary sulfur compounds to sulfuric acid (Schippers and Sand 1999). In the case of elemental sulfur, oxidation is exclusively carried out by microorganisms because this sulfur compound is chemically inert in acidic environments. Consequently, elemental sulfur may accumulate in the course of metal sulfide dissolution if sulfur-oxidizing microorganisms are absent or inhibited. The production of protons (acidity) via sulfuric acid generation from oxidation of reduced sulfur compounds is beneficial since protons are consumed in the initial metal sulfide dissolution reaction of the polysulfide pathway (Fig. 3B).

Furthermore, sulfur oxidizers can influence leaching kinetics in a particular manner. Elemental sulfur may occur suspended as free aggregates and crystals or can form a layer on the metal sulfide surface (Mustin et al. 1993). In the latter case, the electrochemical properties of the metal sulfide surface might change and/or a barrier may be formed, reducing the diffusion rates for ions and oxygen. Both phenomena may negatively influence the leaching kinetics. Leaching rate decreasing sulfur and/or polysulfide layers were observed on acid-soluble sphalerite in the absence of sulfur oxidizers (Fowler and Crundwell 1998). Similar problems are known for chalcopyrite as well (Tanne and Schippers 2021). However, at appropriate redox potentials during bioleaching, inhibiting sulfur layers can be avoided.

## Biochemistry of iron(II) oxidation

Within the last years, more information has been obtained by analysis of the redox chains of aerobic iron(II)-oxidizing bacteria such as *At. ferrooxidans* and *L. ferrooxidans* (Castelle et al. 2008, 2010; Blake and Griff 2012). Although most biochemical details are best known for *At. ferrooxidans*, on the basis of spectroscopic biochemical and “omics” analyses, it can be stated that the iron(II)-oxidizing systems of the other acidophilic iron-oxidizing bacteria are different with respect to the redox components used. There are at least 14 genera able to oxidize iron(II) ions with molecular oxygen as an electron acceptor. Within this diversity of microbial groups, it is not astonishing that different mechanisms exist (Blake and Griff 2012; Bonnefoy and Holmes 2011). It has been postulated that these biochemical differences in the respiratory chains could determine whether *At. ferrooxidans* or *L. ferrooxidans* are the dominant bacteria in various mining habitats such as leach dumps, (underground) ore bodies, or bioreactors (Sand et al. 1992; Schrenk et al. 1998; Rawlings et al. 1999; Rawlings 2002). Consequently, clarification of these oxidizing systems is of industrial importance, as it will affect possible improvements in the use of microbes and/or the design of bioleaching plants. Rawlings et al. (1999) described the phenomenon that, in the case of experiments with *At. ferrooxidans*, iron(II) oxidation was possible only at redox potentials of up to + 850 mV (vs. SHE, here and for all following redox values), whereas with *L. ferrooxidans,* iron(II) oxidation occurred at redox potentials of up to + 950 mV. This finding seems to be related to the fact that the inhibitory concentration of iron(III) ions is much lower for *At. ferrooxidans* (3.1 mM) than for *L. ferrooxidans* (42.8 mM) (Norris et al. 1988). Rusticyanin has a midpoint redox potential of around 680 mV, whereas *Leptospirillum* spp. possesses two cytochromes, Cyt_572_ and Cyt_579_, with more positive redox potential, which are proposed to be its key proteins in aerobic iron(II) oxidation. In biofilms, both of them showed post-translational modifications dependent on the maturation state of the biofilm, and Cyt_579_ showed sequence variants with decreased redox potentials (Singer et al. 2010). The aerobic iron respiratory chain of *L. ferrooxidans* is dominated by the redox status of an abundant cellular cytochrome that had an absorbance peak at 579 nm in the reduced state (Blake and Griff 2012). This helps to explain the increased redox potential at which *L. ferrooxidans* can still oxidize iron(II) ions. For *L. ferrooxidans*, considerably lower oxidation and growth rates on iron(II) ions than for *At. ferrooxidans* were measured in laboratory studies (Sand et al. 1992; Hallmann et al. 1993). However, this seems to be true only for a redox potential below 700 mV (SHE); above this value, *L. ferrooxidans* has a higher iron(II) ions oxidation rate than *At. ferrooxidans*. Although this adaptation to high redox potentials is rather inefficient with respect to energy conservation, it has been proposed to explain the dominance of *Leptospirillum* strains found in commercial bioleaching operations (Rawlings et al. 1999).

In the case of *At. ferrooxidans*, the functionality of the respiratory chain coupling iron(II) oxidation with oxygen reduction has been shown. The respiratory system is flexible, and gene expression can be modulated according to the main energy source and the growth condition (Quatrini et al. 2009). Electrons from iron(II) ions can either be transported along a “downhill” or an “uphill” pathway (Amouric et al. 2010). The first pathway allows for ATP synthesis, whereas the second one allows for the production of reducing power for biosynthetic reactions. The *rus* operon encodes the proteins involved in the “downhill pathway,” which includes two c-type cytochromes (Cyc1 and Cyc2), the blue copper protein rusticyanin (Rus), and an aa3-type cytochrome oxidase (CoxABCD) (Appia-Ayme et al. 1999). The 46 kDa Cyc2, with a midpoint potential of + 560 mV, is located in the outer membrane and functions as the primary electron acceptor in iron(II)-oxidation (Yarzabal et al. 2002, 2004). The electron is then transferred via the periplasmic cupredoxin rusticyanin to a membrane-bound Cyc1 (a dihemic c4-type cytochrome), which hands over the electron to the aa3-type cytochrome oxidase (Yamanaka and Fukumori 1995). It has been shown that these proteins are organized in a supramolecular structure spanning the outer and inner membranes. This supercomplex has been proven to be functional since, after its purification in mild conditions, iron(II) oxidation as well as oxygen reduction activities were present (Castelle et al. 2008). Also, the proteins belonging to the bc1 complex (uphill pathway) as well as the cytochrome Cyc2 (c4 type) have been found in this supercomplex, suggesting a strong physical association of the “uphill and downhill” respiratory chains, where, as stated earlier, Rus is modulating the delivery of electrons to both chains. Recently, the Cyc2 protein structure has been modeled by a combination of methodologies adapted for transmembrane proteins. The model suggests a 16-stranded transmembrane beta-barrel structure spanning the outer membrane. Binding sites for iron and one heme C group were predicted. Docking analysis of the Cyc2 structure with Rus and Cyc1 provided a structural explanation for the presence of a “conducting electron wire” along the three proteins, as electron hopping distances were calculated (Jiang et al. 2021). Rus is an essential component of the electron transport chain in *At. ferrooxidans*. An organism that survives on a substrate with such little energy, such as iron(II) ions, may contain up to 5% of its total cell protein in the form of Rus (Cox and Boxer 1978). With a ΔG of only about − 30 kJ/mol available from iron(II)-oxidation (with oxygen as an electron acceptor at pH 2), *At. ferrooxidans* could not afford to produce several percent of its biomass as Rus if it would not have an important function. It is accepted that Rus, besides its bridging function, acts as electron reservoir in *At. ferrooxidans*. Furthermore, this assumption also explains the redox dependence discussed above. As determined by Ingledew and Cobley (1980), Rus has a midpoint redox potential of + 680 mV. As a consequence, it may take up electrons to become reduced up to potentials of around + 800 mV. This agrees well with data presented by Rawlings et al. (1999), which are also supported by other studies (Meruane et al. 2002). Rus, due to its large concentration, could efficiently take up every electron that becomes available, channeling it into the downhill oxidation pathway. The primary electron acceptor (probably Cyc2) remains oxidized. Consequently, the driving force for iron(II)-oxidation is at maximum (i.e., for a certain iron(II)/iron(III) ratio, the ΔG value of iron(II)-oxidation is highest because the other redox partner, the electron acceptor Cyc2, is fully oxidized). This has the advantage that most electrons available from iron(II) ions can be collected, however, only in the redox range of Rus. This seems to be highly beneficial, especially when using such a low-energy substrate. It must be noted that among the Fe(II)-oxidizing acidithiobacilli, at least two different Fe(II) oxidation pathways exist. One is via RusA, working in *At. ferrooxidans*. The second pathway, present in some *At. ferrooxidans* and *At. ferrivorans* strains, includes a high potential iron-sulfur protein (HiPIP) (Bruscella et al. 2005) and the rusticyanin isoenzyme RusB (Amouric et al. 2010; Bird et al. 2011).

## Biochemistry of sulfur oxidation

Acidophilic bioleaching microorganisms possess several reduced inorganic sulfur compounds (RISCs) oxidation systems, some of them working in parallel. The Sox (sulfur-oxidizing) system consists of a set of dehydrogenases and other proteins, which catalyze the oxidation of sulfide, S^0^, thiosulfate, and sulfite to sulfate, accompanied by subsequent electron transfers through cytochromes (Dahl 2020). The essential proteins for an active SOX system are the periplasmic proteins SoxYZ, SoxB, SoxCD, and SoxXA, which interact with each other (Friedrich et al. 2001; Welte et al. 2009). The Sox multienzyme complex is absent in some acidithiobacilli such as *At. ferrooxidans* and *At. thiooxidans*. In these species, a sulfur dioxygenase (Sdo) has been proposed to catalyze the initial S^0^ oxidation step. It has been shown that only thiol-bound sulfane sulfur atoms (R-SS_n_H) are reactive enough to be oxidized by Sdo (Rohwerder and Sand 2003). By bioinformatic analysis, it has been suggested that the gene cluster *hdrABC* (heterodisulfide reductase) and some accessory proteins found to be conserved in several acidithiobacilli as well as sulfur-oxidizing archaea could catalyze a similar sulfur oxidation reaction as SDO (Quatrini et al. 2009). RISCs oxidation is also driven by enzymes such as sulfide:quinone oxidoreductase (Sqr) (Wakai et al. 2004; Brasseur et al. 2004) and thiosulfate:quinone oxidoreductase (Tqo), which oxidizes thiosulfate in vitro with tetrathionate as product and ferricyanide or decyl ubiquinone (DQ) as electron acceptors (Muller et al. 2004). Tetrathionate can be further degraded to thiosulfate by a tetrathionate hydrolase (Tth) (Kanao et al. 2007). This enzyme has also been found extracellular, being responsible for sulfur globule formation in *At. ferrooxidans*^T^, under conditions of low oxygen levels (Steudel et al. 1987; Kanao et al. 2007; Beard et al. 2011). The sulfur oxygenase reductase (Sor) catalyzes an oxygen-dependent sulfur disproportionation reaction to sulfite and hydrogen sulfide. It does not require external cofactors for activity (Kletzin 1989). No energy conservation occurs during Sor catalysis, but its reaction products are substrates for downstream enzymes. Sor has been studied in detail in the thermoacidophilic archaeum *Acidianus* (Kletzin et al. 2004) and also seems to be an important S-oxidizing enzyme in moderate thermophiles such as *Sulfobacillus* species. Its activity has been measured at temperatures up to 75 ℃ in *S. thermosulfidooxidans*^T^ (Janosch et al. 2015). Interestingly, deep branching acidithiobacilli contain *sor* genes, and transcripts have been detected in *At. caldus*, but their contribution to the overall oxidation of sulfur seems to be low, as Sor enzyme activity has not been successfully measured in acidithiobacilli*.* Its presence may be considered to be a “molecular fossil,” supporting that the ancestors of this taxon were most likely thermophiles (Moya-Beltran et al. 2021).

## Concluding remarks

Bioleaching has become an established technology in the mining industry, and research on further applications has expanded over the last years (reviewed in part B). Over the last decades, the knowledge about the diversity of acidophilic iron- and sulfur-oxidizing bacteria and archaea and their interaction with sulfide minerals in terms of oxidation mechanisms, chemical pathways, the relevance of EPS, and biofilm formation has been expanded greatly. The still missing knowledge about detailed interactions of cells with minerals and among themselves on the molecular level might be gained with gene networks and evolutionary traits, high throughput proteomics, development of recombinant strains, and synthetic biology. With this knowledge, several novel bioleaching applications using acidophiles will be possible as green biotechnology.
