# Correction to: Progress in bioleaching: fundamentals and mechanisms of microbial metal sulfide oxidation—part A

**DOI:** 10.1007/s00253-022-12233-1

**Published:** 2022-10-19

**Authors:** Mario Vera, Axel Schippers, Sabrina Hedrich, Wolfgang Sand

**Affiliations:** 1 Instituto de Ingeniería Biológica Y Médica, Escuelas de Ingeniería, Medicina Y Ciencias Biológicas, Pontificia Universidad Católica de Chile, Santiago, Chile; 2 Departamento de Ingeniería Hidráulica Y Ambiental, Escuela de Ingeniería, Pontificia Universidad Católica de Chile, Santiago, Chile; 3 Bundesanstalt Für Geowissenschaften Und Rohstoffe (BGR), Hannover, Germany; 4 Institute of Biosciences, TU Bergakademie Freiberg, Freiberg, Germany; 5 Faculty of Chemistry, University Duisburg-Essen, Essen, Germany

**Correction to: Applied Microbiology and Biotechnology**

**https://doi.org/10.1007/s00253-022-12168-7**

The published version contains some error in the sequence of the authors.

The correct sequence should be as shown below.

Mario Vera^1,2*^, Axel Schippers^3^, Sabrina Hedrich^4^, Wolfgang Sand^4,5*^

This is being corrected in this publication.

